# A 32-Year-Old Male with Corneal Hydrops

**DOI:** 10.5811/cpcem.25351

**Published:** 2024-11-02

**Authors:** Justin Anderson, Ryan Grinnell, Kristina Domanski, Jamie Baydoun

**Affiliations:** * University of Nevada, Las Vegas, Kirk Kerkorian School of Medicine, Las Vegas, Nevada; † University Medical Center of Southern Nevada, Emergency Department, Las Vegas, Nevada

**Keywords:** corneal hydrops, keratoconus, ocular ultrasound

## Abstract

**Case Presentation:**

A 32-year-old male with a history of left eye keratoconus presented to the emergency department with left eye pain and blurry vision for two days. Out of concern for corneal hydrops, ophthalmology was consulted, and the diagnosis was confirmed. Per ophthalmology recommendations, the patient was started on hypertonic saline and prednisolone eye drops and referred to a corneal specialist.

**Discussion:**

Corneal hydrops is characterized by stromal edema caused by leakage of aqueous humor due to rupture of Descemet membrane. This case describes a patient with a keratoconus deformity who developed corneal hydrops.

## CASE PRESENTATION

**Image 1:**
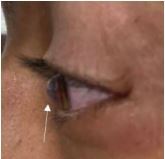
Sagittal view of the left eye showing a bulging of the cornea consistent with keratoconus deformity, as indicated by the arrow.

**Image 2:**
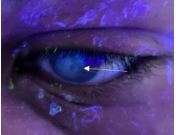
Fluorescein staining did not show any uptake including over the opacification, identified by the arrow.

**Image 3:**
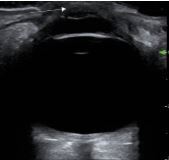
Ocular ultrasound shows keratoconus deformity identified by the white arrow.

A 32-year-old male with a history of left eye keratoconus secondary to remote trauma presented to the emergency department (ED) with left eye pain and blurry vision for two days. Visual acuity was 20/40 in the right eye and 20/200 in the left eye, and 20/25 bilaterally with baseline corrective lenses. Examination showed central left eye corneal opacification overlying the pupil and keratoconus deformity (Image 1). Fluorescein exam revealed no uptake over the pupil (Image 2). Ocular ultrasound showed a deformed cornea (Image 3).

## DISCUSSION

Corneal hydrops is characterized by stromal edema caused by leakage of aqueous humor due to rupture of Descemet membrane.^1^ It is a rare complication of keratoconus, likely due to a combination of corneal thinning and ectasia and trivial trauma to the eye.^2^ Risk factors include atopy, Down syndrome, keratoconus, and eye rubbing, which may incur the highest risk. Acute corneal hydrops can cause vision-debilitating scarring of the cornea.^3^ The ED workup of suspected corneal hydrops should rule out infectious causes of corneal edema such as keratitis and uveitis, as well as include a fluorescein exam to rule out corneal ulcer. Treatment is usually conservative, and most cases resolve within two to four months.

Given our patient’s history of keratoconus, with new onset opacification of the cornea and without fluorescein uptake, the presentation was concerning for corneal hydrops. An ocular ultrasound was performed, which revealed the keratonoconus deformity, but it was otherwise unremarkable, with normal optic nerve sheath, lens, iris, and without abnormal retinal contour. Ophthalmology was consulted, and the diagnosis was confirmed. The patient was started on 5% sodium chloride eye drops and prednisolone eye drops per ophthalmology recommendations to decrease edema, and he was referred for urgent outpatient follow-up with a corneal specialist.

Initial outpatient management of corneal hydrops centers around decreasing the edema and includes options such as antibiotics to prevent secondary infection, hypertonic saline drops to cause an osmotic gradient to reduce edema, cycloplegics for pain control, and topical nonsteroidal anti-inflammatory drugs/steroids to decrease inflammation and pain.^1^ Surgery is sometimes indicated and can improve visual acuity and delay corneal transplantation.^4^ When ophthalmology is not on call, medical management is as discussed, and transfer to a facility with ophthalmology should be considered, as the patient will require urgent outpatient follow-up with ophthalmology as surgical intervention may be required.^4^

**CPC-EM Capsule**

What do we already know about this clinical entity?

*Corneal hydrops is a rare entity that can cause severe corneal damage and permanent blindness without intervention.*

What is the major impact of the image(s)?

*Clinically it is similar in appearance to corneal abrasions or ulcerations, but it has no fluorescein uptake on staining.*

How might this improve emergency medicine practice?

*Corneal hydrops must be identified early and managed appropriately to avoid risk of significant morbidity.*
